# Disease accumulation and distribution across the lifespan in Swedish centenarians and non-centenarians: a nationwide life course comparison of longevity and health resilience

**DOI:** 10.1016/j.eclinm.2025.103396

**Published:** 2025-08-02

**Authors:** Yuge Zhang, Shunsuke Murata, Katharina Schmidt-Mende, Marcus Ebeling, Karin Modig

**Affiliations:** a Unit of Epidemiology, Institute of Environmental Medicine, Karolinska Institutet, Stockholm, Sweden; b Academic Primary Health Care Centre, Stockholm Region, Stockholm, Sweden; c Division of Family Medicine and Primary Care, Department of Neurobiology, Care Sciences and Society, Karolinska Institutet, Huddinge, Sweden; d Max Planck Institute for Demographic Research, Rostock, Germany

**Keywords:** Centenarians, Multimorbidity, Prevalence, Birth cohort, Longevity, Health resilience

## Abstract

**Background:**

Previous research suggests that centenarians reach exceptional ages primarily by avoiding major diseases rather than surviving them. However, how they manage multiple conditions over the life course remains less understood. Examining the accumulation and distribution of diseases across lifespan can provide insights into mechanisms underlying their resilience.

**Methods:**

We conducted a nationwide historical prospective study including all individuals born in Sweden between 1920 and 1922 (n = 274,108), tracking their health from age 70 for up to 30 years. Disease trajectories of centenarians were compared to those of shorter-lived peers using national health registers. We analysed disease burden, the rate of disease accumulation, and patterns of multimorbidity across age groups.

**Findings:**

Centenarians had fewer diagnosed conditions and accumulated diseases at a slower rate than non-centenarians. Cardiovascular diseases were the most common diagnoses in all age groups, but contributed less to the overall disease burden among centenarians. In contrast, malignancies accounted for a relatively larger share of their disease profile. Neuropsychiatric conditions were consistently less common among centenarians, showing the largest relative difference across all ages. Centenarians also had fewer co-occurring diseases and were more likely to have conditions confined to a single disease group.

**Interpretation:**

Our findings of a lower overall disease burden, a delayed onset of multiple conditions, and fewer co-occurring diseases over time among centenarians (compared to non-centenarians) suggest a preserved homeostatic capacity and sustained functional integrity in the face of cumulative physiological stressors. Future research should aim to identify genetic, epigenetic, and environmental factors underlying these patterns to inform early-life preventive strategies that promote longevity and resilience.

**Funding:**

10.13039/501100004047Karolinska Institutet.

**Research in context**

**Evidence before this study**

We systematically searched PubMed and Web of Science using the terms “(centenarian) AND ((disease trajectory) OR (disease accumulation) OR (multimorbid∗) OR (disease pattern))” and “ALL = (centenarian) AND ALL = (“disease pattern” OR “multimorbidity” OR “disease trajectory” OR “disease accumulation”)” from database inception to March 31, 2025, without language restrictions. Some previous studies have found that centenarians tend to have fewer chronic diseases than individuals who die in their 80s or 90s. However, few have examined differences in the rate of disease accumulation, and existing findings are inconsistent. A few studies have used cluster analysis to explore multimorbidity patterns among centenarians and individuals dying at different ages. Most of this research has either focused only on centenarians or made comparisons with non-centenarians at different chronological ages, often based on cross-sectional data or short follow-up periods. To fully understand how diseases accumulate across the life course, it is ideal to compare centenarians with individuals born in the same period. However, longitudinal data enabling such comparisons are rare. While one study suggests that centenarians are more likely to avoid major diseases compared to non-centenarians from the same birth cohort, no prior research has used a full birth cohort design to examine whether both severe and non-severe conditions accumulate differently between centenarians and non-centenarians at comparable ages across the life span.

**Added value of this study**

To our knowledge, this is the first study to comprehensively compare the accumulation of diseases, contributions of specific conditions to multimorbidity, and disease combination patterns between centenarians and non-centenarians from the same birth cohorts including institutionalised individuals over a 30-year follow-up period. Our findings show that centenarians follow a distinct trajectory of disease accumulation, characterised by fewer conditions and slower progression to multimorbidity at every age. In contrast to non-centenarians, whose disease burden accelerates sharply in the final years of life, centenarians exhibit a stabilisation in disease accumulation from their 90s onward. They also have fewer co-occurring conditions and are more likely to present with diseases confined to a single disease group at comparable ages. Notably, lower prevalence and a delayed onset of cardiovascular diseases appear to be key contributors to their exceptional longevity. In addition, centenarians demonstrate a superior resistance to neuropsychiatric diseases throughout life.

**Implications of all the available evidence**

The study reveals that centenarians are more likely to have diseases confined to a single diagnostic group, suggesting that multimorbidity patterns—and not just the number of conditions—may be crucial in predicting long-term survival. The relative resistance to cardiovascular and neuropsychiatric diseases among centenarians highlights potential targets for translational research. Since differences in disease accumulation begin to diverge long before extreme old age, preventive strategies may be most effective when implemented in midlife or earlier. This underscores the importance of life-course approaches to chronic disease prevention. Finally, the slower progression of disease and lower overall disease burden among centenarians suggests that extreme longevity may not be as resource-intensive as commonly assumed. These insights can support more nuanced planning of long-term care services by distinguishing the needs of the oldest old. Future research should explore the underlying biological and social determinants of centenarians’ enhanced resilience, to guide targeted interventions that promote longevity and support healthy ageing trajectories.

## Introduction

Centenarians, individuals who survive to at least 100 years of age, represent a highly selective group characterised by exceptional longevity. However, their number and share of the population have increased significantly over time. Previous research indicates that centenarians reach their exceptional age by avoiding, and to some extent delaying, the onset of major diseases rather than surviving them to a greater extent.^1^^,^^2^ This challenges the assumption that longer lifespans inevitably lead to higher disease rates. Instead, centenarians represent a distinct group with exceptional disease resilience.^2^^,^^3^ However, these findings are based on single, life-threatening conditions such as stroke and myocardial infarction, which typically represent the final stages of broader disease processes. As such, centenarians’ apparent resilience may reflect the ability to avoid severe disease manifestations rather than the complete absence of underlying pathology. The degree of resilience may, therefore, differ when broader and more common conditions—such as hypertension or atrial fibrillation—are considered. It is plausible that centenarians do not avoid these milder or earlier-stage conditions to the same extent, but instead manage them more effectively or prevent their progression. To gain a more comprehensive understanding of disease resilience in extreme old age, it is essential to examine the full trajectory of disease accumulation, including multiple coexisting conditions. This approach can clarify whether centenarians develop underlying diseases without advancing to the same level of complexity as their shorter-lived peers, thereby offering deeper insight into the mechanisms supporting healthy longevity.

Studies analysing and comparing disease accumulation across ages remain rare, with only a few addressing this topic.^4^^,^^5^ Most existing studies have examined multimorbidity (co-occurrence of diseases) cross-sectionally,^6^^,^^7^ yet ageing is a dynamic process in which the contribution and combination of diseases evolve over time. Consequently, multimorbidity patterns identified at a single point in time or over short follow-up periods may fail to capture the long-term progression of disease accumulation and coexistence.^7^^,^^8^ To understand how multiple diseases develop over the lifespan, it is essential to compare disease accumulations over longer periods of time at comparable ages between representative centenarians and non-centenarians minimising distortions from period and cohort effects. Moreover, many studies exploring disease accumulation in centenarians are limited by small, local samples or the exclusion of institutionalised individuals, potentially affecting the generalisability of their findings.^4^, ^5^, ^6^, ^7^^,^^9^

In this study, we investigated how disease accumulation differs over the life course between centenarians and non-centenarians from the same birth cohorts using historical prospective data from nationwide administrative health records. Specifically, we examined whether centenarians exhibit similar but delayed disease accumulation, and whether this delay is eventually followed by a steeper increase in later life. Moreover, whether the patterns of multimorbidity differ between centenarians and non-centenarians, for example whether centenarians accumulate different or less severe diseases, and if their disease combinations are different. Through this, we aim to provide deeper insights into the ageing process and determinants of exceptional longevity.

## Methods

### Study design, population, and ethics

All individuals born in 1920, 1921, and 1922 who were alive and residing in Sweden at age 70 years were identified in the Total Population Register. Individuals were followed prospectively from January 1, 1990, until their death, 100th birthday, or December 31, 2022, whichever came first, enabling follow-up of all individuals from age 70 to potentially 100 years. Individuals who emigrated after age 70 were excluded from the study population (n = 2003, 0.73%). Mortality selection of the birth cohorts can be found in Supplementary Figure S1. This study was performed in line with the principles of the Declaration of Helsinki. This study was approved by the regional ethics committee in Stockholm (Dnr 2011/136–31/5 and amendment 2022-03486-02). The ethics board waived the need for written informed consent from participants.

### Disease definition

Through a unique personal identification number, data on all inpatient (hospital) and outpatient (specialist) care visits, along with corresponding International Classification of Diseases (ICD) -diagnosis codes, were linked to each individual from the National Patient Register. Diseases considered in multimorbidity research are highly heterogeneous, which poses challenges for cross-study comparisons.^10^ To address this, we used a previously adopted disease list to analyse multimorbidity patterns.^5^^,^^11^^,^^12^ We adopted the same conditions and disease groupings but added four additional age-related diseases which are particularly common among individuals of exceptional age.^13^, ^14^, ^15^, ^16^, ^17^ In total, 40 conditions were included in the analysis. These 40 conditions were grouped into 10 disease categories: anaemia, cardiovascular, digestive, endocrine, malignancy, neuropsychiatric, musculoskeletal, neurosensorial, respiratory, and urological diseases. Each group comprised a varying number of specific conditions. Both primary and secondary diagnoses from hospital records were used to identify diseases between the 9th and 10th revisions of the ICD codes. This approach aimed to maximise the identification of prevalent conditions across the study population. More details can be found in Supplementary Table S1.

The number and combinations of diseases, multimorbidity, were described from age 70 and onwards. A three-year period prior to age 70 was used to identify diseases at age 70 in the register. As individuals aged, the tracking period extended correspondingly, continuing until their age at death or end of follow-up, meaning that centenarian trajectories are based on 30 years of follow-up. Only the first occurrence of each specific disease was considered, meaning that once a disease was identified at a certain age, its presence remained coded as 1 for all subsequent ages. This approach enabled a longitudinal capture of how multimorbidity accumulated over the life course.

### Statistical analysis

First, the accumulation of specific diseases from age 70 onwards was calculated for each age in the total population and in subgroups based on age at death, presented as means and medians. Percentile bootstrap methods with 1000 resamples were applied to calculate the 95% confidence interval (95% CI). The average annual increase was calculated to assess the rate of disease accumulation. The proportion of the population with 0 to >5 diseases by age at death was also calculated as well as the distribution of number of diseases at different ages earlier in life for people with different lifespans was further explored.

Next, we analysed which diseases contributed to disease accumulation at different ages, depending on lifespan. This was done by calculating the absolute contribution (mean number) as well as relative contribution (proportion) of the 10 disease groups to the total average number of diseases at various ages and by age at death. Each disease group contained the accumulation of all specific diseases within that category. For instance, if an individual had hypertension and heart failure at age 70, they contributed two diseases to the cardiovascular (CVD) group. If, by age 80, they also developed a myocardial infarction, their contribution to the CVD group increased to three diseases. This analysis also allows for the observation of changes in the absolute and relative contributions of disease groups from one age to another in centenarians and those who died earlier.

Finally, we characterised and compared the prevalence and the specific combinations of disease groups at comparable ages earlier in life between centenarians and non-centenarians.^18^ Notably, unlike previous calculations, because of complexity, disease combinations were described at the group level only, meaning that individuals could have one or more diseases within a given disease group.

Exploratory post hoc analyses were performed following descriptive analyses. We restricted our comparisons to reduce multiple statistical tests and focused on the most relevant differences. Welch's t-test were used to compare the absolute contribution of each disease group to the total average number of diseases, and Chi-square tests were used to evaluate differences in proportion of individuals whose diseases were confined to a single group between centenarians and non-centenarians at age 80, as age 80 is a representative midpoint across the life span spectrum, approximate to the average lifespan. All statistical tests were two-sided with a significance of *p* < 0.05. Given the potential sex differences in morbidity patterns and survival, sex-specific analyses were conducted as sensitivity analyses to examine whether the accumulation of diseases and disease contribution patterns were consistent across sexes. All analyses were conducted using SAS 9.4 (SAS Institute Inc., Cary, NC, USA) and R (version 4.4.2).

### Role of the funding source

The funder had no role in study design, data collection, data analysis, data interpretation, or writing of the report.

## Results

The study included 274,108 individuals, of whom 46.2% were male and 53.8% were female. Among them, 1.6% reached the age of 100. Table 1 provides a description of the study population by age at death. At the time of death, 72.7% had more than one disease, 66.8% had diseases from at least two disease groups, and 12.6% had no record of any of the included diseases.

**Table 1:** Characteristics of study population, total and stratified by age at death, aged 70 years and above in Sweden.

	Dying between ages 70–79 (n = 81,493)	Dying between ages 80–89 (n = 119,829)	Dying between ages 90–99 (n = 68,456)	Dying ≥ age 100 (n = 4330)	Total (n = 274,108)
Number of females, (%)	34,016 (41.7)	64,456 (53.8)	45,506 (66.5)	3500 (80.8)	147,478 (53.8)
Number of disease group at the end of follow-up (%)					
0	22,101 (27.1)	10,117 (8.4)	2185 (3.2)	151 (3.5)	34,554 (12.6)
1	26,282 (32.3)	22,396 (18.7)	7227 (10.6)	445 (10.3)	56,350 (20.6)
≥2	33,110 (40.6)	87,316 (72.9)	59,044 (86.2)	3734 (86.2)	183,204 (66.8)
Number of individual diseases at the end of follow-up (%)					
0	22,101 (27.1)	10,117 (8.4)	2185 (3.2)	151 (3.5)	34,554 (12.6)
1	19,772 (24.3)	15,671 (13.1)	4650 (6.8)	310 (7.2)	40,403 (14.7)
≥2	39,620 (48.6)	94,041 (78.5)	61,621 (90.0)	3869 (89.3)	199,151 (72.7)

Note: The end of follow-up is the date of death or the date of turning age 100.

**Fig. 1:**
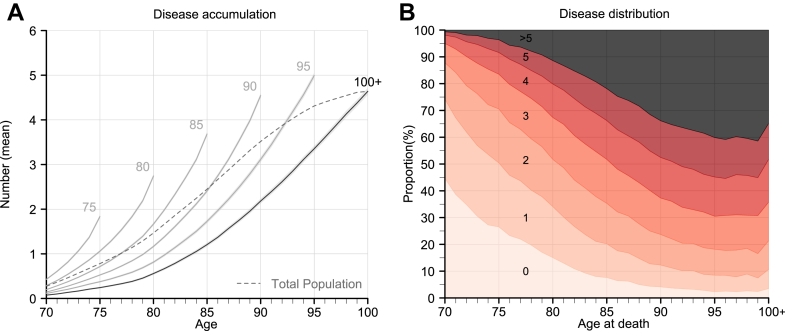
Disease accumulation from age 70 (A) and proportion of individuals with 0 to >5 diseases by age at death (B), birth cohorts 1920–1922, Sweden. Note: The numbers by each line in panel (A) represent age at death. The numbers in different coloured areas in panel (B) represent numbers of diseases.

Fig. 1 presents the absolute (A) and relative (B) cumulative accumulation of individual diseases. Panel A illustrates that centenarians had a lower mean number of diseases at every age compared to non-centenarians. For example, at age 85, centenarians had on average 1.2 diagnosed diseases, whereas people dying at age 90 had on average 2.4 diagnosed diseases. The pattern was consistent for both males and females (Supplementary Figure S5). Despite living the longest, the average number of diseases among centenarians never exceeded that of individuals who died in their 90s (Similar in medians, Supplementary Figure S2). While the rate of disease accumulation increased with age for all groups, centenarians experienced a slower accumulation rate at comparable ages. In the last 10 years of life, disease accumulation remained stable in centenarians, whereas it continued to rise in individuals with shorter lifespans (Supplementary Table S2).

Panel B presents the proportion of diseases by age at death. Individuals with shorter lifespans exhibited a right skewed distribution of diseases, indicating that most did not experience a high disease burden. For instance, 26.5% of individuals who died at age 75 had no diagnosed diseases, whereas the corresponding proportion among centenarians was only 3.5%. The pattern of disease distribution by age at death was consistent for both males and females (Supplementary Figure S5). While the number of diseases increased with age, the distribution of diseases varied depending on lifespan. For example, at age 70, 50% of the individuals, regardless of lifespan, had 0 diseases, but the share of individuals having more was higher at earlier ages at death (Supplementary Figure S3). By age 90, 80% of centenarians had a maximum of 5 diseases with a median of 2, compared to a maximum of 6 diseases and a median of 3 among those who died at age 95 (Supplementary Figure S3).

**Fig. 2:**
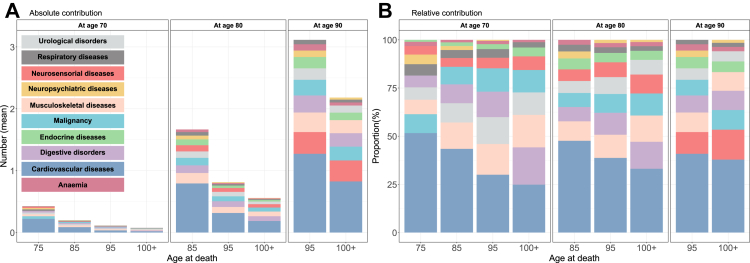
The absolute (A) and relative (B) contribution of different disease groups to the average number of diseases at ages 70, 80 and 90 for individuals with different lifespans (x-axis), birth cohorts 1920–1922, Sweden. Note: Diseases are presented in descending order, ranked from highest to lowest in terms of composition and proportion, from bottom to top.

Fig. 2 illustrates the absolute (A) and relative (B) contributions of different diseases (within groups) to the mean number of diseases at age 70, 80 and 90 years for centenarians and for individuals who died at age 75, 85 and 95 years respectively. While centenarians had a similar disease group composition at each comparable age, their absolute disease burden remained lower than that of individuals who died earlier, with similar pattern in both males and females (Supplementary Figures S6 and S7). The slower rate of disease accumulation observed in Fig. 1 applied to all disease groups, as shown in Fig. 2. However, the relative contributions of some disease groups declined with age, including respiratory and musculoskeletal diseases. Cardiovascular diseases were the most prevalent disease group at all ages, regardless of lifespan, in both absolute and relative terms. The second most common disease group varied by age and lifespan: malignancies were most common for those dying at age 75, musculoskeletal diseases for those dying at age 85, and musculoskeletal diseases (at ages 70 and 80) and neurosensorial diseases (at age 90) for those dying at age 95. Among centenarians, digestive disorders were the second most prevalent disease group at ages 70 and 80, whereas neurosensorial diseases became the most common by age 90. The contribution of specific disease groups varied slightly by sex. Among males, urological disorders consistently ranked as the second most common disease group across nearly all ages. In contrast, among females, musculoskeletal diseases were the second most prevalent for those who died at ages 85 and 95, and also for centenarians at age 80. Further details are provided in Supplementary Tables S3, S5, S7 and S8.

While CVD contributed the most to the mean number of diseases in all groups, centenarians had the lowest CVD burden at comparable ages compared to individuals who died earlier. At age 70, CVD accounted for 51.6% of the total disease burden among individuals who died at age 75, more than twice the proportion observed in centenarians (24.8%) at age 70. At age 80, CVD comprised 47.6% of diseases in those who died at age 85, 38.7% in those who died at age 90, and 33.1% in centenarians. By age 90, CVD represented 40.8% of diseases among those who died at age 95 and 37.8% in centenarians. The difference in CVD burden between centenarians and non-centenarians narrowed with age, and the relative contribution of CVD increased more rapidly between ages 80 and 90 in centenarians than in non-centenarians (Supplementary Table S4).

In contrast to CVD, neuropsychiatric diseases consistently contributed the least among centenarians across ages. Both in absolute and relative terms, their contribution was lower compared to non-centenarians, with the gap widening at older ages. Additionally, centenarians exhibited a higher proportion of malignancies than non-centenarians at ages 80 and 90. This pattern was consistent in both males and females. Further details are provided in Supplementary Tables S3–S5, S7–S10.

**Fig. 3:**
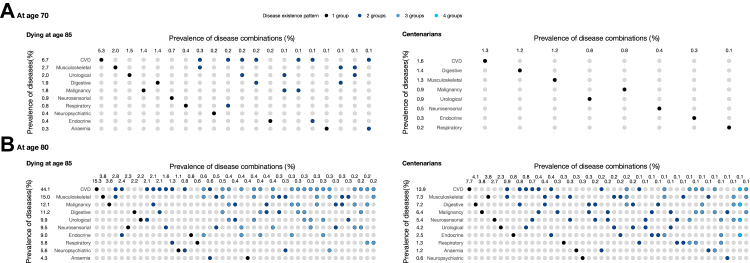
Prevalence of disease groups (y-axis) and prevalence of combinations of disease groups (x-axis) at ages 70 (A) and 80 (B) for individuals reaching average lifespan (dying at age 85) and becoming centenarians, birth cohorts 1920–1922, Sweden. Note: The x-axis represents the prevalence of the 45 most common disease existence patterns, ranked in descending order. Disease existence patterns are displayed using dots in different colours (single disease in black, 2 in dark blue, 3 in grey blue, 4 in sky blue). Only disease combination with a prevalence of 0.1% and higher are included in the figure.

While Fig. 2 illustrates the contribution of different disease groups to the average number of diseases, it does not depict the extent of co-occurrence among diseases within the same individuals. Fig. 3 addresses this by presenting disease combinations at age 70 and 80 for centenarians and for individuals who died at age 85 (with results for age 90 and other ages at death available in Supplementary Figure S4). The figure shows the prevalence of individuals with only one disease group, as well as those with different combinations of disease groups. The y-axis represents the prevalence of disease groups, either alone or in combination, while the x-axis shows the prevalence of each disease group, both alone and in specific combinations. The data are sorted from highest to lowest prevalence and only include diseases with combination prevalences of 0.1% or higher. Neuropsychiatric diseases consistently had the lowest prevalence among centenarians compared to all other disease groups. Moreover, the relative difference in the prevalence of neuropsychiatric conditions between centenarians and those who died at younger ages was the largest across all age groups.

At age 70, centenarians exhibited a low prevalence of all diseases, with no disease combination exceeding a prevalence of 0.1%. For individuals dying at age 85, it was most common to have only one disease, though several combinations of disease groups were present with a prevalence above 0.1%. The most common combination was CVD and musculoskeletal diseases, with a prevalence of 0.3%. The most prevalent disease group was CVD, with a prevalence of 1.6% among centenarians and 6.7% among those dying at age 85. The majority of individuals had only CVD, with a prevalence of 1.3% for centenarians and 5.3% for those dying at age 85.

By age 80, it became more common for centenarians to have diseases from multiple groups. The prevalence of CVD had risen to 13.9% among centenarians and 44.1% among individuals dying at age 85. The prevalence of having only CVD was 7.7% among centenarians and 15.3% among those dying at age 85, indicating that by this age, two-thirds of individuals dying at age 85 had more than just CVD, while approximately half of centenarians had only CVD. Further details on the proportion of individuals with diseases confined to a single group are presented in Supplementary Table S6. The most common disease combination was CVD and musculoskeletal diseases, observed in 0.9% of centenarians and 2.8% of individuals dying at age 85. The second most prevalent combination was CVD and digestive disorders (0.8%) among centenarians, and CVD and endocrine diseases (2.4%) among those dying at age 85. The most common three-disease combination was CVD, musculoskeletal diseases, and digestive disorders for centenarians (0.2%), and CVD, musculoskeletal diseases, and endocrine diseases for individuals dying at age 85 (0.6%).

## Discussion

This study is the first to compare disease accumulation across a wide range of conditions with varying severity over time in centenarians and non-centenarians from the same birth cohorts. The primary aim was to investigate whether centenarians exhibit delayed disease accumulation, and a distinct disease panorama compared to their shorter-lived peers. The findings indicate that centenarians experience a lower number of diseases and a slower rate of disease accumulation at every age, resulting in a delayed progression to multimorbidity. While the overall disease burden is shaped by similar conditions in both centenarians and non-centenarians, their relative contributions differ. Centenarians have a lower relative burden of cardiovascular disease but a relatively higher contribution of some other conditions such as malignancies. The patterns of disease combinations are also largely similar; however, centenarians are more likely to have conditions within a single disease group.

Previous research has observed that centenarians exhibit lower levels of multimorbidity, measured by the number of diseases,^4^, ^5^, ^6^, ^7^^,^^9^ mostly based on cross sectional studies.^6^^,^^7^ Our findings extend this observation by demonstrating that the slower disease accumulation among centenarians is a continuous process evident from age 70 onward. Moreover, centenarians not only differ from individuals who die prematurely but also exhibit delayed disease accumulation compared to those who live beyond the average lifespan. Another notable finding is that disease accumulation remained stable in the final years of life for centenarians, whereas it continued to rise in individuals with shorter lifespans. This aligns with a German study that reported a less steep increase in comorbidities among centenarians compared to non-centenarians.^9^ However, it contrasts findings from a Swedish and a U.S. study suggesting the rate of disease accumulation to be steeper than that of non-centenarians.^4^^,^^5^ This discrepancy may be due to differences in study design, as the latter studies were based on smaller samples with less detailed follow-up data. Additionally, centenarians and non-centenarians in those studies were drawn from different birth cohorts, meaning centenarians were born earlier, which could bias comparisons by making their health status appear worse and their disease accumulation appear faster had they been compared to individuals from the same birth cohorts and at comparable ages during the same calendar time. Furthermore, we used both primary and secondary diagnoses to maximise the detection of diseases and improve sensitivity in identifying multimorbidity patterns. Since our analysis only included the first recorded occurrence of each disease, regardless of the order of the diagnosis code, there is no risk of overestimating the prevalence from a potential over-coding of secondary diagnoses.

Different disease groups may reflect distinct mechanisms associated with longevity. Cardiovascular diseases represent the greatest disease burden throughout life for both centenarians and non-centenarians, yet centenarians consistently exhibit a lower contribution of CVD across all ages. This advantage has been documented in previous research,^1^^,^^2^^,^^19^^,^^20^ with centenarians showing both a lower risk and delayed onsets of CVD. A similar delay in onset of CVD has also been observed among the offspring of centenarians compared to the offspring of non-centenarians,^20^ suggesting a potential inheritable protective effect.^21^ However, previous studies have primarily focused on specific diseases rather than examining how a lower CVD burden influences the overall disease accumulation over time. The lower levels of CVD at all ages both in absolute and relative terms suggest that CVD is one of the key drivers of the slower disease accumulation in centenarians.

In contrast to CVD, centenarians exhibit a relatively larger contribution of malignancies to the total disease burden than non-centenarians, particularly at ages 80 and 90. This finding may suggest that centenarians do not completely avoid cancer but may be able to mitigate its impact, or develop less aggressive forms of cancer.^22^ They may have greater resistance to cancer progression or respond more effectively to treatment.^23^ The underlying mechanisms for this resilience remain unclear. Since inherited genetic factors have a more limited influence on many types of malignancy compared to cardiometabolic diseases,^21^ future research should explore the biological and environmental factors contributing to centenarians' ability to withstand cancer. However, it is important to note that despite a relatively higher contribution of malignancy to disease burden in centenarians, the overall prevalence of malignancies remains lower in centenarians compared to non-centenarians.

We observed that neuropsychiatric diseases, including dementia and depression, contributed the least to the total disease burden among centenarians, with the largest prevalence gap between centenarians and non-centenarians at comparable ages. Dementia is one of the severe and burdensome diseases in old age and a major cause of disability and mortality.^24^ It has been expected to become the leading cause of death in the near future instead of CVD with 5-year mortality risk of more than 50%.^25^ Our findings suggest centenarians’ exceptional lifespans can be related to resilience to neuropsychiatric diseases, as they appear to avoid it to a higher extent than non-centenarians at comparable ages earlier in life. While this aligns with previous studies showing how centenarians have managed to maintain high levels of cognitive functioning,^19^^,^^26^ the overall prevalence of neuropsychiatric diseases appears somewhat lower in our study than in previous research.^24^^,^^27^ This is probably because we identify disease based on diagnoses in hospital and outpatient specialist care, and miss diagnoses set in primary care only.^24^ However, as the same approach was applied for both centenarians and non-centenarians, the underestimation is likely nondifferential and does not compromise the observed patterns.

Studies of exceptionally long lived, such as centenarians, and their health trajectories are essential for advancing our understanding of the dynamics of health and physiological resilience across the life course. Our findings of a lower overall disease burden, a delayed onset of multiple conditions and fewer co-occurring diseases over time suggest a preserved homeostatic capacity and sustained functional integrity in the face of cumulative physiological stressors.^28^ Importantly, this disease resilience, when compared to non-centenarians, becomes apparent well before extreme old age, emerging as early as age 70, indicating that their distinctive health profiles likely originate earlier in life. The underlying mechanisms of this resilience are plausibly shaped by a favourable combination of genetic, epigenetic, and environmental influences. Previous research has shown that centenarians possess a comparable number of disease-associated genetic variants as their shorter-lived peers, but they appear to delay the onset and progression of disease.^29^^,^^30^ This may be attributed to gene–environment interactions that attenuate the deleterious effects of pathogenic variants or to the presence of longevity-associated genetic polymorphisms that mitigate the impact of harmful mutations.^1^^,^^31^ Nevertheless, the critical question of when and how, across the life course, the interaction between genetic predisposition and lifestyle factors begins to diverge between centenarians and non-centenarians remains unresolved. Our findings challenge the prevailing assumption that advanced chronological age is inherently accompanied by a substantial disease burden or inevitable physiological decline.

Given that divergence in health trajectories between centenarians and non-centenarians arises relatively early in adulthood, the results highlight the imperative of initiating preventive strategies and targeted interventions in midlife to preserve physiological resilience. Cardiovascular diseases, in particular, emerge as a key differentiating domain. These conditions typically progress insidiously before manifesting as more severe events, such as myocardial infarction or stroke. Centenarians appear more capable of avoiding or delaying this progression, though the extent to which this is achieved through advantageous lifestyle behaviours, genetic endowment, or intensified secondary prevention remains to be determined.

Going forward, the early identification of homeostatic capacity—potentially via composite biomarker panels and the development of appropriate age-specific clinical thresholds—will likely be critical for informing future clinical practice. Tailoring such approaches could facilitate more precise risk stratification and intervention strategies aimed at promoting healthy longevity.

The primary strength of this study lies in its use of unique data comprising 30 years of longitudinal and comprehensive disease records for an entire population, minimising selection bias. Additionally, the study design, which compares centenarians and non-centenarians from the same birth cohorts, reduces distortions caused by period and cohort effects. However, certain limitations should be acknowledged. First, diseases were identified based on healthcare visits recorded in the National Patient Register, which may lead to an underestimation of true disease prevalence. This underestimation is likely consistent across all individuals in the study. Nevertheless, if centenarians and their shorter-lived peers systematically exhibit different healthcare-seeking behaviours, this could introduce some degree of over- or underestimation in the observed differences. Moreover, it might be possible that not every diagnostic procedure is pursued in the oldest old. However, given Sweden's publicly funded and nearly free healthcare system as well as performing well on care quality,^32^^,^^33^ such systematic biases are unlikely to substantially distort the overall patterns observed. Second, the analysis of disease combinations was based on disease groups rather than individual diseases, and the 40 individual diseases were not evenly distributed across the 10 disease groups. Consequently, the higher contribution of certain disease groups to multimorbidity may partly reflect the larger number of individual diseases included within these groups. However, since centenarians and non-centenarians were analysed using the same approach, any potential bias introduced by this classification was likely mitigated. Additionally, while individual diseases within the same group may vary in their contribution, previous research has demonstrated that diseases within a given group tend to exhibit homogeneity, and group-based analyses remain consistent across various health outcomes.^34^^,^^35^

Our findings highlight the distinct health trajectories of centenarians, emphasising their ability to delay multimorbidity and maintain a lower disease burden compared to their shorter-lived peers. This delay occurs across almost all disease types, though particularly lower levels and a delayed onset of cardiovascular diseases appear to play a key role in their exceptional longevity. Additionally, centenarians experience fewer co-occurring diseases, especially at younger ages, and are more likely to have conditions confined to a single disease group. This pattern may reflect both biological resilience and potential protective mechanisms against certain diseases. Identifying genetic, epigenetic, or environmental factors associated with these patterns could lead to novel interventions for promoting longevity and resilience.

## Contributors

KM, ME and YZ conceptualised the study. KM was responsible for data acquisition and funding. YZ, SM, KSM, ME and KM accessed and verified the underlying data. SM contributed to methodology. YZ analysed the data with the supervision of all co-authors. KSM contributed to the validation of particularly medical diagnoses. YZ drafted the first version of the manuscript. All authors contributed substantially to the study design, data interpretation, and critical revisions of the manuscript. All authors were responsible for the final approval of the manuscript. All listed authors meet authorship criteria.

## Data sharing statement

The individual level data underlying this study cannot be shared publicly because of the General Data Protection Regulation in Sweden. Access to the data and statistical code can be permitted to external researchers after ethical vetting and establishment of a collaboration agreement. Contact the corresponding author for questions about data sharing.

## Declaration of interests

YZ declares support from internal KID funding, a block grant for partial funding of doctoral education from Karolinska Institutet. KM declares research project grant from Swedish Research Council for Health, Working Life and Welfare (FORTE) and being a board member of the National Screening Board at the Swedish National Board of Health and Welfare during the past 36 months. All other authors declare no competing interests.

## References

[1] Ismail K., Nussbaum L., Sebastiani P. (2016). Compression of morbidity is observed across cohorts with exceptional longevity. J Am Geriatr Soc.

[2] Zhang Y., Murata S., Schmidt-Mende K., Ebeling M., Modig K. (2025). Do people reach 100 by surviving, delaying, or avoiding diseases? A life course comparison of centenarians and non-centenarians from the same birth cohorts. Geroscience.

[3] Willcox D.C., Willcox B.J., Wang N.C., He Q., Rosenbaum M., Suzuki M. (2008). Life at the extreme limit: phenotypic characteristics of supercentenarians in Okinawa. J Gerontol A Biol Sci Med Sci.

[4] Ailshire J.A., Beltrán-Sánchez H., Crimmins E.M. (2015). Becoming centenarians: disease and functioning trajectories of older US Adults as they survive to 100. J Gerontol A Biol Sci Med Sci.

[5] Vetrano D.L., Grande G., Marengoni A., Calderón-Larrañaga A., Rizzuto D. (2021). Health trajectories in Swedish centenarians. J Gerontol A Biol Sci Med Sci.

[6] Clerencia-Sierra M., Ioakeim-Skoufa I., Poblador-Plou B. (2020). Do centenarians die healthier than younger Elders? A comparative epidemiological study in Spain. J Clin Med.

[7] Ioakeim-Skoufa I., Clerencia-Sierra M., Moreno-Juste A. (2022). Multimorbidity clusters in the oldest old: results from the EpiChron cohort. Int J Environ Res Public Health.

[8] Gellert P., von Berenberg P., Zahn T., Neuwirth J., Kuhlmey A., Dräger D. (2019). Multimorbidity profiles in German centenarians: a latent class analysis of health insurance data. J Aging Health.

[9] Gellert P., von Berenberg P., Oedekoven M. (2018). Centenarians differ in their comorbidity trends during the 6 years before death compared to individuals who died in their 80s or 90s. J Gerontol A Biol Sci Med Sci.

[10] Calderón-Larrañaga A., Vetrano D.L., Onder G. (2017). Assessing and measuring chronic multimorbidity in the older population: a proposal for its operationalization. J Gerontol A Biol Sci Med Sci.

[11] Marengoni A., Rizzuto D., Wang H.X., Winblad B., Fratiglioni L. (2009). Patterns of chronic multimorbidity in the elderly population. J Am Geriatr Soc.

[12] Rizzuto D., Melis R.J.F., Angleman S., Qiu C., Marengoni A. (2017). Effect of chronic diseases and multimorbidity on survival and functioning in elderly adults. J Am Geriatr Soc.

[13] Gibson W., Wagg A. (2014). New horizons: urinary incontinence in older people. Age Ageing.

[14] Miller A.P., Huff C.M., Roubin G.S. (2016). Vascular disease in the older adult. J Geriatr Cardiol.

[15] Leung F.W., Rao S.S.C. (2009). Fecal incontinence in the elderly. Gastroenterol Clin.

[16] Schöön I.M., Mellström D., Odén A., Ytterberg B.O. (1989). Incidence of peptic ulcer disease in Gothenburg, 1985. BMJ.

[17] Ahsberg K., Ye W., Lu Y., Zheng Z., Staël von Holstein C. (2011). Hospitalisation of and mortality from bleeding peptic ulcer in Sweden: a nationwide time-trend analysis. Aliment Pharmacol Ther.

[18] Lex A., Gehlenborg N. (2014). Sets and intersections. Nat Methods.

[19] Andersen S.L., Sebastiani P., Dworkis D.A., Feldman L., Perls T.T. (2012). Health span approximates life span among many supercentenarians: compression of morbidity at the approximate limit of life span. J Gerontol A Biol Sci Med Sci.

[20] Terry D.F., Wilcox M.A., McCormick M.A., Perls T.T. (2004). Cardiovascular disease delay in centenarian offspring. J Gerontol A Biol Sci Med Sci.

[21] van den Berg N., Rodríguez-Girondo M., van Dijk I.K., Slagboom P.E., Beekman M. (2023). Increasing number of long-lived ancestors marks a decade of healthspan extension and healthier metabolomics profiles. Nat Commun.

[22] Andersen S.L., Terry D.F., Wilcox M.A., Babineau T., Malek K., Perls T.T. (2005). Cancer in the oldest old. Mech Ageing Dev.

[23] Yao S., Boudreau R.M., Galvin A. (2024). All-cause mortality and cause-specific death in U.S. long-lived siblings: data from the long life family study. J Gerontol A Biol Sci Med Sci.

[24] Ding M., Ek S., Aho E., Jönsson L., Schmidt-Mende K., Modig K. (2024). Prevalence of dementia diagnosis in Sweden by geographical region and sociodemographic subgroups: a nationwide observational study. Lancet Reg Health Eur.

[25] van de Vorst I.E., Vaartjes I., Geerlings M.I., Bots M.L., Koek H.L. (2015). Prognosis of patients with dementia: results from a prospective nationwide registry linkage study in the Netherlands. BMJ Open.

[26] Beker N., Sikkes S.A.M., Hulsman M. (2020). Longitudinal maintenance of cognitive health in centenarians in the 100-plus study. JAMA Netw Open.

[27] Qiu C., von Strauss E., Bäckman L., Winblad B., Fratiglioni L. (2013). Twenty-year changes in dementia occurrence suggest decreasing incidence in central Stockholm, Sweden. Neurology.

[28] Fried L.P., Cohen A.A., Xue Q.-L., Walston J., Bandeen-Roche K., Varadhan R. (2021). The physical frailty syndrome as a transition from homeostatic symphony to cacophony. Nat Aging.

[29] Caruso C., Ligotti M.E., Accardi G. (2022). How important are genes to achieve longevity?. Int J Mol Sci.

[30] Freudenberg-Hua Y., Freudenberg J., Vacic V. (2014). Disease variants in genomes of 44 centenarians. Mol Genet Genomic Med.

[31] Beekman M., Blanché H., Perola M. (2013). Genome-wide linkage analysis for human longevity: genetics of healthy aging study. Aging Cell.

[32] Anell A. (2015). The public–private pendulum — patient choice and equity in Sweden. N Engl J Med.

[33] Ludvigsson J.F., Bergman D., Lundgren C.I. (2025). The healthcare system in Sweden. Eur J Epidemiol.

[34] Vetrano D.L., Roso-Llorach A., Fernández S. (2020). Twelve-year clinical trajectories of multimorbidity in a population of older adults. Nat Commun.

[35] Vetrano D.L., Rizzuto D., Calderón-Larrañaga A. (2018). Trajectories of functional decline in older adults with neuropsychiatric and cardiovascular multimorbidity: a Swedish cohort study. PLoS Med.

