# Supplementary material for: Disease accumulation and distribution across the lifespan in Swedish centenarians and non-centenarians: a nationwide life course comparison of longevity and health resilience
Source: eClinicalMedicine. 2025 Aug 2;87:103396. doi: 10.1016/j.eclinm.2025.103396 (PMC12341696; doi:10.1016/j.eclinm.2025.103396)
Supplement: Supplementary Materials [file mmc1.docx]

**Supplementary materials**

**Disease accumulation and distribution across the lifespan in Swedish centenarians and non-centenarians: a nationwide life course comparison of longevity and health resilience**

Yuge Zhang, ^a^ Shunsuke Murata, ^a^ Katharina Schmidt-Mende, ^b,c^ Marcus Ebeling, ^a,d^ Karin Modig, ^a^

^a^Unit of Epidemiology, Institute of Environmental Medicine, Karolinska Institutet, Stockholm, Sweden

^b^Academic Primary Health Care Centre, Stockholm Region, Stockholm, Sweden

^c^Division of Family Medicine and Primary Care, Department of Neurobiology, Care Sciences and Society, Karolinska Institutet, Huddinge, Sweden

^d^Max Planck Institute for Demographic Research, Rostock, Germany

**Corresponding author:** Yuge Zhang, Institute of Environmental Medicine, Karolinska Institutet, Box 210, 17177 Stockholm, Sweden; Email: [yuge.zhang@ki.se](mailto:yuge.zhang@ki.se)

**Table of Contents**

[Table S1 The Swedish 9^th^ and 10^th^ revisions of the International Classification of Diseases (ICD) codes used to identify diseases 3](#_Toc203182914)

[Figure S1 Mortality selection of birth cohorts 1920-1922, Sweden 5](#_Toc203182915)

[Table S2 The rate of disease accumulation per age from age 70 6](#_Toc203182916)

[Figure S2 Disease accumulation from age 70 for individuals with different lifespans, birth cohorts 1920-1922, Sweden 7](#_Toc203182917)

[Figure S3 Disease distribution at ages 70, 80 and 90 for individuals with different lifespans (x-axis), birth cohorts 1920-1922, Sweden 8](#_Toc203182918)

[Table S3 Changes in absolute contribution (mean number) of disease groups for individuals with different lifespans, birth cohorts 1920-1922, Sweden 9](#_Toc203182919)

[Table S4 Changes in relative contribution (proportion) of disease groups for individuals with different lifespans, birth cohorts 1920-1922, Sweden 11](#_Toc203182920)

[Table S5 Comparison of absolute contribution (mean number) of disease groups between centenarians and those dying at age 85 or 95 at age 80 13](#_Toc203182921)

[Figure S4 Prevalence of diseases (y-axis) and prevalence of disease combinations (x-axis) at ages 70, 80 and 90 for individuals with different life spans, birth cohorts 1920-1922, Sweden 14](#_Toc203182922)

[Table S6 The share of having diseases confined in only one group at age 80 15](#_Toc203182923)

[Figure S5 Disease accumulation from age 70 and proportion of individuals with 0 to >5 diseases by age at death for male (A) and female (B), birth cohorts 1920-1922, Sweden 16](#_Toc203182924)

[Figure S6 The absolute (A) and relative (B) contribution of different disease groups to the average number of diseases at ages 70, 80 and 90 for male with different lifespans (x-axis), birth cohorts 1920-1922, Sweden 17](#_Toc203182925)

[Figure S7 The absolute (A) and relative (B) contribution of different disease groups to the average number of diseases at ages 70, 80 and 90 for female with different lifespans (x-axis), birth cohorts 1920-1922, Sweden 18](#_Toc203182926)

[Table S7 Changes in absolute contribution (mean number) of disease groups for male with different lifespans, birth cohorts 1920-1922, Sweden 19](#_Toc203182927)

[Table S8 Changes in absolute contribution (mean number) of disease groups for female with different lifespans, birth cohorts 1920-1922, Sweden 21](#_Toc203182928)

[Table S9 Changes in relative contribution (proportion) of disease groups for male with different lifespans, birth cohorts 1920-1922, Sweden 23](#_Toc203182929)

[Table S10 Changes in relative contribution (proportion) of disease groups for female with different lifespans, birth cohorts 1920-1922, Sweden 25](#_Toc203182930)

Table S1 The Swedish 9^th^ and 10^th^ revisions of the International Classification of Diseases (ICD) codes used to identify diseases

| **Disease groups** | **Individual diseases** | **ICD−10** | **ICD−9** |
| --- | --- | --- | --- |
| Anaemia | Anaemia | D50-D64 | 280-285 |
| Cardiovascular diseases | Chronic rheumatic heart disease | I05**−**09 | 393-398 |
|  | Heart failure | I50 | 428 |
|  | Atrial fibrillation | I48 | 427D |
|  | Hypertension | I10−15 | 401-405 |
|  | Ischemic heart disease | I20−25 | 410-414 |
|  | Cerebrovascular diseases | I60−69, G45 | 430-438 |
|  | Cardiomyopathy | I42, I43 | 425 |
| Digestive disorders | Intestinal diverticula | K57 | 562 |
|  | Chronic liver disease | K70−77 | 571, 572, 573, 570 |
|  | Functional digestive disorders | K59 | 564F, 564G, 564H, 564W, 564X |
|  | Cholelithiasis | K80 | 574 |
| Endocrine diseases | Thyroid dysfunction | E00−07 | 240-246 |
|  | Diabetes | E10−14 | 259 |
| Malignancy | Malignant neoplasms | C00−97 | 140-208 |
| Neuropsychiatric diseases | Depression | F32; F33; F34; F38; F39 | 296B, 296W, 296X, 298A, 300E, 300X, 301B, 311 |
|  | Dementia | F00-F03, F05.1, G30, G31.1, G31.8A, G31.9 | 290, 294B, 331A-331C, 331X |
|  | Schizophrenia | F20−21 | 295A, 295B, 295C, 295D, 295F, 295G, 295W, 295X |
|  | Alcohol dependence syndrome | F10.2 | 303 |
| Musculoskeletal diseases | Osteoporosis | M80−81 | 733A, 733B |
|  | Osteoarthrosis | M15 | 715 |
|  | Rheumatoid arthritis | M05−06, M12.3 | 714A-714C, 714W, 714X, 719D |
|  | Hip fracture | S72 | 820 |
|  | Crystal arthropathies | M10 | 274, 984X |
|  | Polymyalgia rheumatica | M35.3 | 725 |
| Neurosensorial diseases | Deafness, hearing impairments | H80, H90, H91.1, H91.3, H91.9 | 387, 389, 388A |
|  | Visual impairments or glaucoma | H54, H40 | 369, 365A-D, 365F-X |
|  | Migraine | G43 | 346 |
|  | Peripheral neuropathies | G50 | 350-359 |
|  | Parkinson disease | G20 | 332A |
|  | Epilepsy | G40 | 345,333C |
| Respiratory diseases | Chronic obstructive pulmonary diseases | J40−47 | 490-494, 496 |
|  | Pneumoconiosis | J60−66 | 500-505 |
| Urological disorders | Renal calculus | N20 | 592 |
|  | Prostate hypertrophy | N40 | 600 |
|  | Renal failure | N18, N19 | 585-586 |
| **Additional added 4 conditions** | | | |
| **Disease groups** | **Individual diseases** | **ICD−10** | **ICD−9** |
| Cardiovascular diseases | Peripheral vascular disease | I70, I71, I73.1, I73.8, I73.9, I77.1, I79.0, I79.2, K55 | 440, 441, 443B, 443W, 443X, 447B, 557 |
| Digestive disorders | Peptic ulcer disease | K25-K28 | 531-534 |
| Urological disorders | Urine incontinence | N39.3, N39.4, R32 | 625G, 788D |
| Digestive disorders | Faecal incontinence | R15 | 787G |


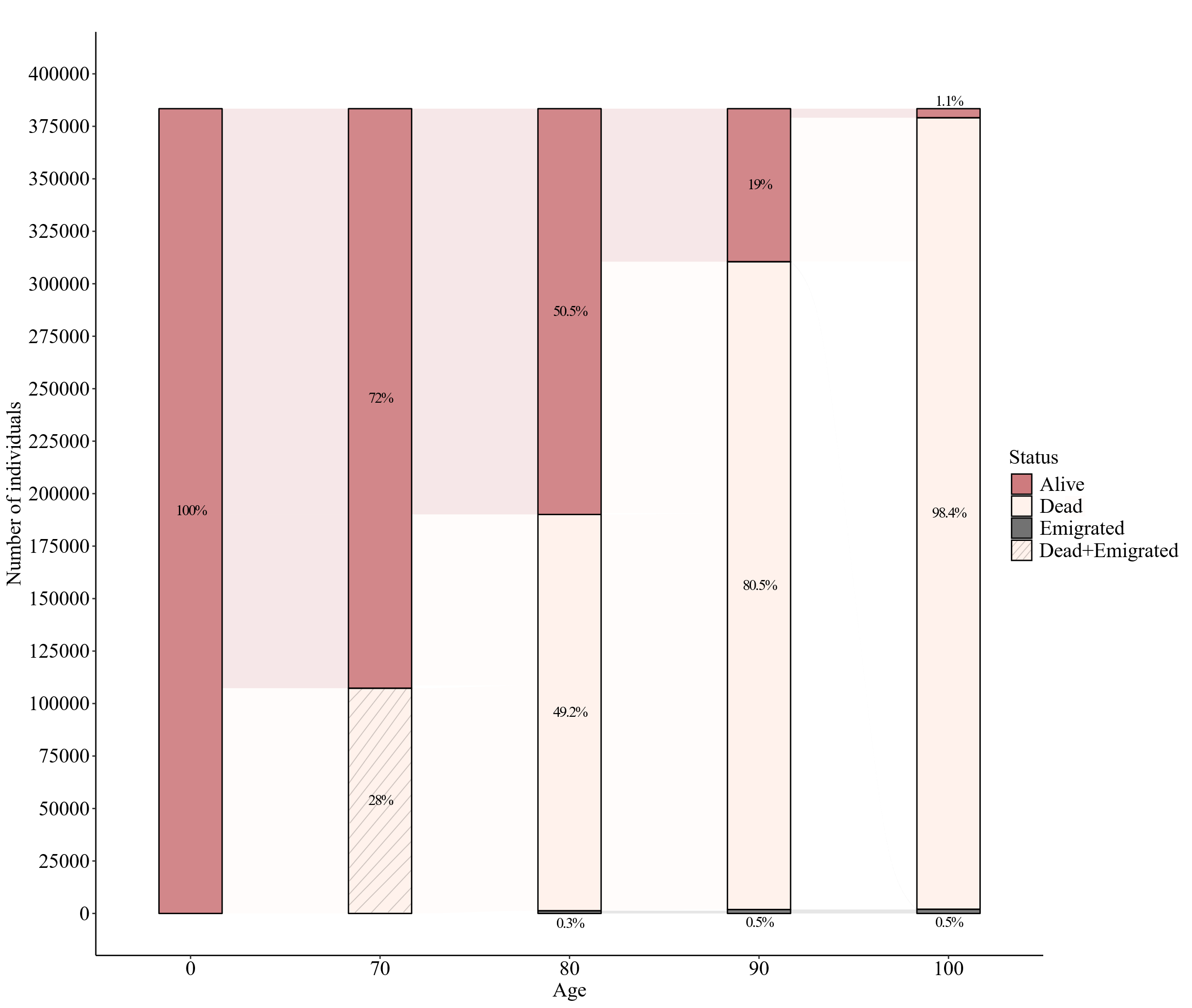


Figure S1 Mortality selection of birth cohorts 1920-1922, Sweden

Note: A total of 383,422 individuals were born in Sweden between 1920-1922. 107,311 (28%) individuals died or emigrated before age 70, leaving 276,111 (72%) individuals alive and residing in Sweden at age 70. A total of 274,108 individuals (99.27% of those alive at age 70) were followed from age 70 until death or reaching age 100.

The study population consisted of individuals alive and residing in Sweden at age 70, excluding individuals who emigrated out of Sweden after this age (0.73% of those alive at age 70).

Table S2 The rate of disease accumulation per age from age 70

| **Age** | **Dying at age 75** | **Dying at age 80** | **Dying at age 85** | **Dying at age 90** | **Dying at age 95** | **Centenarians** |
| --- | --- | --- | --- | --- | --- | --- |
| 70 | 0 | 0 | 0 | 0 | 0 | 0 |
| 71 | 0.18 | 0.11 | 0.08 | 0.06 | 0.05 | 0.03 |
| 72 | 0.21 | 0.14 | 0.09 | 0.07 | 0.05 | 0.04 |
| 73 | 0.25 | 0.16 | 0.10 | 0.08 | 0.06 | 0.03 |
| 74 | 0.30 | 0.17 | 0.11 | 0.08 | 0.06 | 0.04 |
| 75 | 0.47 | 0.20 | 0.12 | 0.09 | 0.05 | 0.03 |
| 76 | / | 0.22 | 0.14 | 0.09 | 0.06 | 0.04 |
| 77 | / | 0.26 | 0.16 | 0.11 | 0.07 | 0.04 |
| 78 | / | 0.30 | 0.18 | 0.12 | 0.08 | 0.05 |
| 79 | / | 0.36 | 0.20 | 0.14 | 0.10 | 0.07 |
| 80 | / | 0.55 | 0.27 | 0.18 | 0.12 | 0.10 |
| 81 | / | / | 0.31 | 0.21 | 0.16 | 0.11 |
| 82 | / | / | 0.35 | 0.23 | 0.17 | 0.12 |
| 83 | / | / | 0.38 | 0.25 | 0.18 | 0.13 |
| 84 | / | / | 0.41 | 0.26 | 0.19 | 0.13 |
| 85 | / | / | 0.57 | 0.29 | 0.22 | 0.15 |
| 86 | / | / | / | 0.34 | 0.24 | 0.16 |
| 87 | / | / | / | 0.37 | 0.25 | 0.20 |
| 88 | / | / | / | 0.42 | 0.27 | 0.19 |
| 89 | / | / | / | 0.45 | 0.30 | 0.20 |
| 90 | / | / | / | 0.56 | 0.32 | 0.23 |
| 91 | / | / | / | / | 0.33 | 0.21 |
| 92 | / | / | / | / | 0.37 | 0.22 |
| 93 | / | / | / | / | 0.37 | 0.24 |
| 94 | / | / | / | / | 0.38 | 0.26 |
| 95 | / | / | / | / | 0.43 | 0.24 |
| 96 | / | / | / | / | / | 0.25 |
| 97 | / | / | / | / | / | 0.26 |
| 98 | / | / | / | / | / | 0.27 |
| 99 | / | / | / | / | / | 0.24 |
| 100 | / | / | / | / | / | 0.26 |


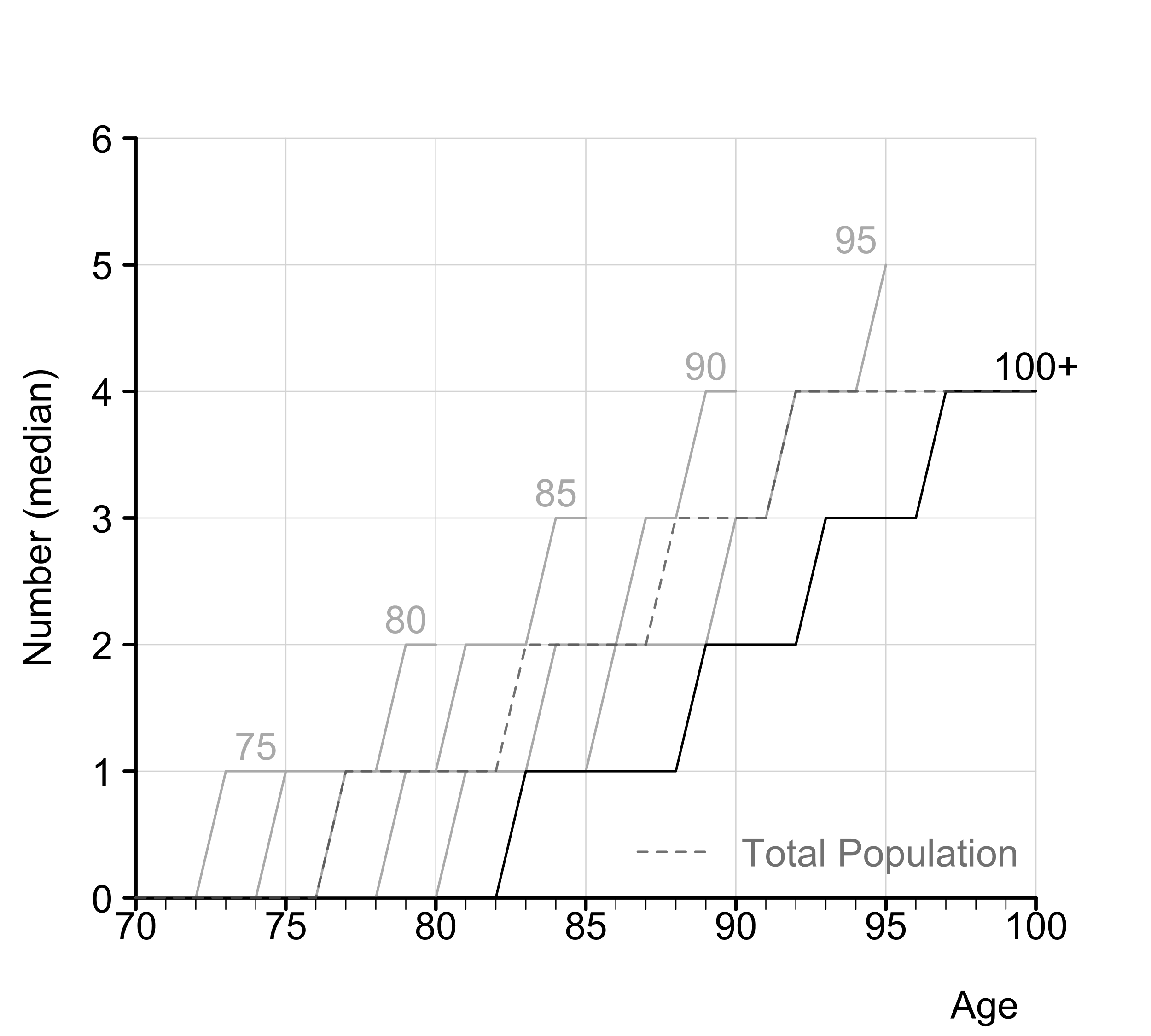


Figure S2 Disease accumulation from age 70 for individuals with different lifespans, birth cohorts 1920-1922, Sweden

Note: The numbers by each line represent age at death.


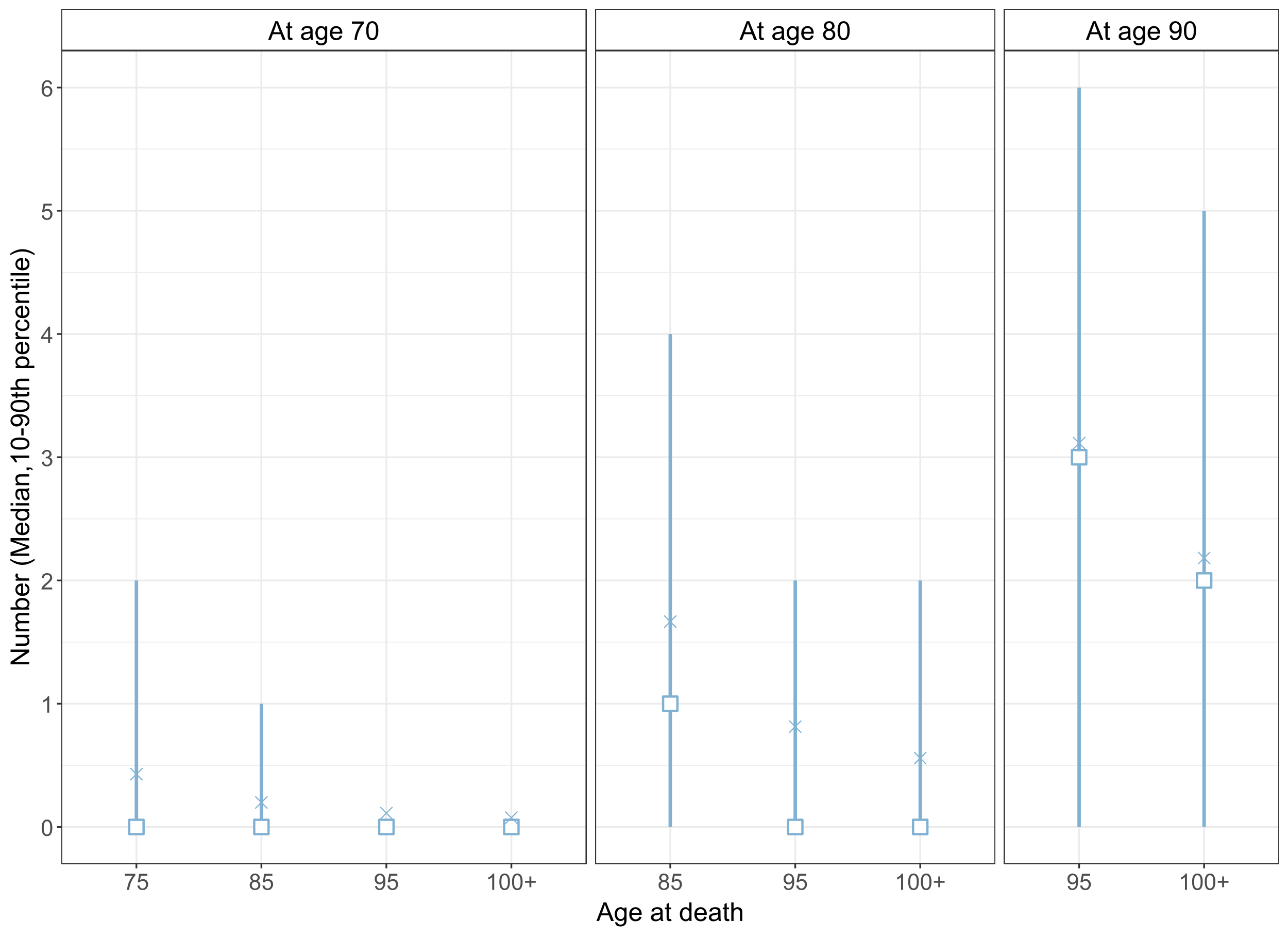


Figure S3 Disease distribution at ages 70, 80 and 90 for individuals with different lifespans (x-axis), birth cohorts 1920-1922, Sweden

Note: The square represents median, and cross represents mean. The upper and lower end of each line represent the 10^th^ and the 90^th^ percentiles.

Table S3 Changes in absolute contribution (mean number) of disease groups for individuals with different lifespans, birth cohorts 1920-1922, Sweden

|  | **Disease groups** | **Mean at age 70** | **Mean at age 80 (average change per age year from age 70 to 80)** | **Mean at age 90 (average change per age year from age 80 to 90)** |
| --- | --- | --- | --- | --- |
| Dying at age 75 | Anaemia | 0.009 | - | - |
|  | Endocrine | 0.004 | - | - |
|  | Neuropsychiatric | 0.021 | - | - |
|  | Respiratory | 0.025 | - | - |
|  | Neurosensorial | 0.019 | - | - |
|  | Malignancy | 0.042 | - | - |
|  | Digestive | 0.026 | - | - |
|  | Urological | 0.027 | - | - |
|  | Musculoskeletal | 0.032 | - | - |
|  | CVD | 0.221 | - | - |
|  | Total | 0.426 | - | - |
| Dying at age 85 | Anaemia | 0.003 | 0.043 (0.004) | - |
|  | Endocrine | 0.004 | 0.094 (0.009) | - |
|  | Neuropsychiatric | 0.004 | 0.060 (0.006) | - |
|  | Respiratory | 0.008 | 0.058 (0.005) | - |
|  | Neurosensorial | 0.009 | 0.100 (0.009) | - |
|  | Malignancy | 0.018 | 0.121 (0.010) | - |
|  | Digestive | 0.019 | 0.124 (0.010) | - |
|  | Urological | 0.020 | 0.104 (0.008) | - |
|  | Musculoskeletal | 0.027 | 0.169 (0.014) | - |
|  | CVD | 0.086 | 0.793 (0.071) | - |
|  | Total | 0.20 | 1.67 (0.147) | - |
| Dying at age 95 | Anaemia | 0.002 | 0.018 (0.002) | 0.102 (0.008) |
|  | Endocrine | 0.003 | 0.039 (0.004) | 0.182 (0.014) |
|  | Neuropsychiatric | 0.001 | 0.014 (0.001) | 0.104 (0.009) |
|  | Respiratory | 0.005 | 0.024 (0.002) | 0.072 (0.005) |
|  | Neurosensorial | 0.006 | 0.062 (0.006) | 0.352 (0.029) |
|  | Malignancy | 0.014 | 0.080 (0.007) | 0.253 (0.017) |
|  | Digestive | 0.015 | 0.093 (0.008) | 0.276 (0.018) |
|  | Urological | 0.016 | 0.071 (0.006) | 0.185 (0.011) |
|  | Musculoskeletal | 0.018 | 0.098 (0.008) | 0.316 (0.022) |
|  | CVD | 0.034 | 0.315 (0.028) | 1.272 (0.096) |
|  | Total | 0.11 | 0.81 (0.07) | 3.11 (0.23) |
| Centenarians | Anaemia | 0.001 | 0.012 (0.001) | 0.049 (0.004) |
|  | Endocrine | 0.003 | 0.026 (0.002) | 0.121 (0.096) |
|  | Neuropsychiatric | 0 | 0.006 (0.001) | 0.034 (0.003) |
|  | Respiratory | 0.002 | 0.013 (0.001) | 0.046 (0.003) |
|  | Neurosensorial | 0.005 | 0.055 (0.005) | 0.339 (0.028) |
|  | Malignancy | 0.009 | 0.064 (0.006) | 0.222 (0.016) |
|  | Digestive | 0.015 | 0.078 (0.006) | 0.218 (0.014) |
|  | Urological | 0.009 | 0.042 (0.003) | 0.115 (0.007) |
|  | Musculoskeletal | 0.013 | 0.076 (0.006) | 0.218 (0.014) |
|  | CVD | 0.019 | 0.185 (0.02) | 0.825 (0.064) |
|  | Total | 0.08 | 0.56 (0.048) | 2.18 (0.162) |

Note: Average change per age year between age 70 and 80 is calculated as: (mean number at age 80-mean number at age 70)/10.

Similarly, average change per age year between age 80 and 90 is calculated as: (mean number at age 90-mean number at age 80)/10.

Table S4 Changes in relative contribution (proportion) of disease groups for individuals with different lifespans, birth cohorts 1920-1922, Sweden

|  | **Disease groups** | **Proportion at age 70 (%)** | **Proportion at age 80 (%) (average change per age year from age 70 to 80)** | **Proportion at age 90 (%) (average change per age year from age 80 to 90)** |
| --- | --- | --- | --- | --- |
| Dying at age 75 | Anaemia | 2.2 | - | - |
|  | Endocrine | 1.0 | - | - |
|  | Neuropsychiatric | 5.0 | - | - |
|  | Respiratory | 5.9 | - | - |
|  | Neurosensorial | 4.5 | - | - |
|  | Malignancy | 9.8 | - | - |
|  | Digestive | 6.1 | - | - |
|  | Urological | 6.4 | - | - |
|  | Musculoskeletal | 7.5 | - | - |
|  | CVD | 51.6 | - | - |
| Dying at age 85 | Anaemia | 1.3 | 2.6 (0.13) | - |
|  | Endocrine | 2.0 | 5.7 (0.37) | - |
|  | Neuropsychiatric | 2.0 | 3.6 (0.16) | - |
|  | Respiratory | 4.1 | 3.5 (-0.06) | - |
|  | Neurosensorial | 4.6 | 6.0 (0.14) | - |
|  | Malignancy | 9.1 | 7.3 (-0.18) | - |
|  | Digestive | 9.8 | 7.4 (-0.24) | - |
|  | Urological | 10.0 | 6.2 (-0.38) | - |
|  | Musculoskeletal | 13.7 | 10.1 (-0.36) | - |
|  | CVD | 43.4 | 47.6 (0.42) | - |
| Dying at age 95 | Anaemia | 1.7 | 2.2 (0.05) | 3.3 (0.11) |
|  | Endocrine | 2.5 | 4.8 (0.23) | 5.9 (0.11) |
|  | Neuropsychiatric | 0.6 | 1.7 (0.11) | 3.3 (0.16) |
|  | Respiratory | 4.5 | 3.0 (-0.15) | 2.3 (-0.07) |
|  | Neurosensorial | 5.5 | 7.7 (0.22) | 11.3 (0.36) |
|  | Malignancy | 12.1 | 9.8 (-0.23) | 8.1 (-0.17) |
|  | Digestive | 13.2 | 11.4 (-0.18) | 8.9 (-0.25) |
|  | Urological | 13.9 | 8.7 (-0.52) | 6.0 (-0.27) |
|  | Musculoskeletal | 15.9 | 12.0 (-0.39) | 10.1 (-0.19) |
|  | CVD | 30.0 | 38.7 (0.87) | 40.8 (0.21) |
| Centenarians | Anaemia | 1.2 | 2.2 (0.1) | 2.3 (0.01) |
|  | Endocrine | 4.6 | 4.7 (0.01) | 5.5 (0.08) |
|  | Neuropsychiatric | 0 | 1.2 (0.12) | 1.6 (0.04) |
|  | Respiratory | 2.8 | 2.4 (-0.04) | 2.1 (-0.03) |
|  | Neurosensorial | 7.1 | 9.8 (0.27) | 15.5 (0.57) |
|  | Malignancy | 11.7 | 11.5 (-0.02) | 10.2 (-0.13) |
|  | Digestive | 19.3 | 14.0 (-0.53) | 10.0 (-0.4) |
|  | Urological | 11.7 | 7.6 (-0.41) | 5.3 (-0.23) |
|  | Musculoskeletal | 16.9 | 13.6 (-0.33) | 9.7 (-0.39) |
|  | CVD | 24.8 | 33.1 (0.83) | 37.8 (0.47) |

Note: Average change per age year between age 70 and 80 is calculated as: (proportion at age 80-proportion at age 70)/10.

Similarly, average change per age year between age 80 and 90 is calculated as: (proportion at age 90-proportion at age 80)/10.

Table S5 Comparison of absolute contribution (mean number) of disease groups between centenarians and those dying at age 85 or 95 at age 80

| **Disease groups** | **Centenarians** | **Dying at age 85** | **Dying at age 95** | ***P (age 100, 85)*** | ***P (age 100, 95)*** |
| --- | --- | --- | --- | --- | --- |
| Anaemia | 0.012 | 0.043 | 0.018 | 1.92E-35 | 0.018 |
| Endocrine | 0.026 | 0.094 | 0.039 | 8.44E-73 | 0.0003 |
| Neuropsychiatric | 0.006 | 0.060 | 0.014 | 2.66E-93 | 0.0003 |
| Respiratory | 0.013 | 0.058 | 0.024 | 3.23E-59 | 3.62E-05 |
| Neurosensorial | 0.055 | 0.100 | 0.062 | 1.26E-23 | 0.101 |
| Malignancy | 0.064 | 0.121 | 0.080 | 1.42E-33 | 0.002 |
| Digestive | 0.078 | 0.124 | 0.093 | 8.41E-17 | 0.011 |
| Urological | 0.042 | 0.104 | 0.071 | 1.16E-46 | 4.35E-10 |
| Musculoskeletal | 0.076 | 0.169 | 0.098 | 4.57E-58 | 0.0002 |
| CVD | 0.185 | 0.793 | 0.315 | 0.00E+00 | 2.82E-29 |

Note: *P(age 100, 85)* represents comparison between centenarians and individuals dying at age 85. Similarly, *P (age 100, 95)* represents comparison between centenarians and individuals dying at age 95. *P*<0.05 was considered statistically significant.


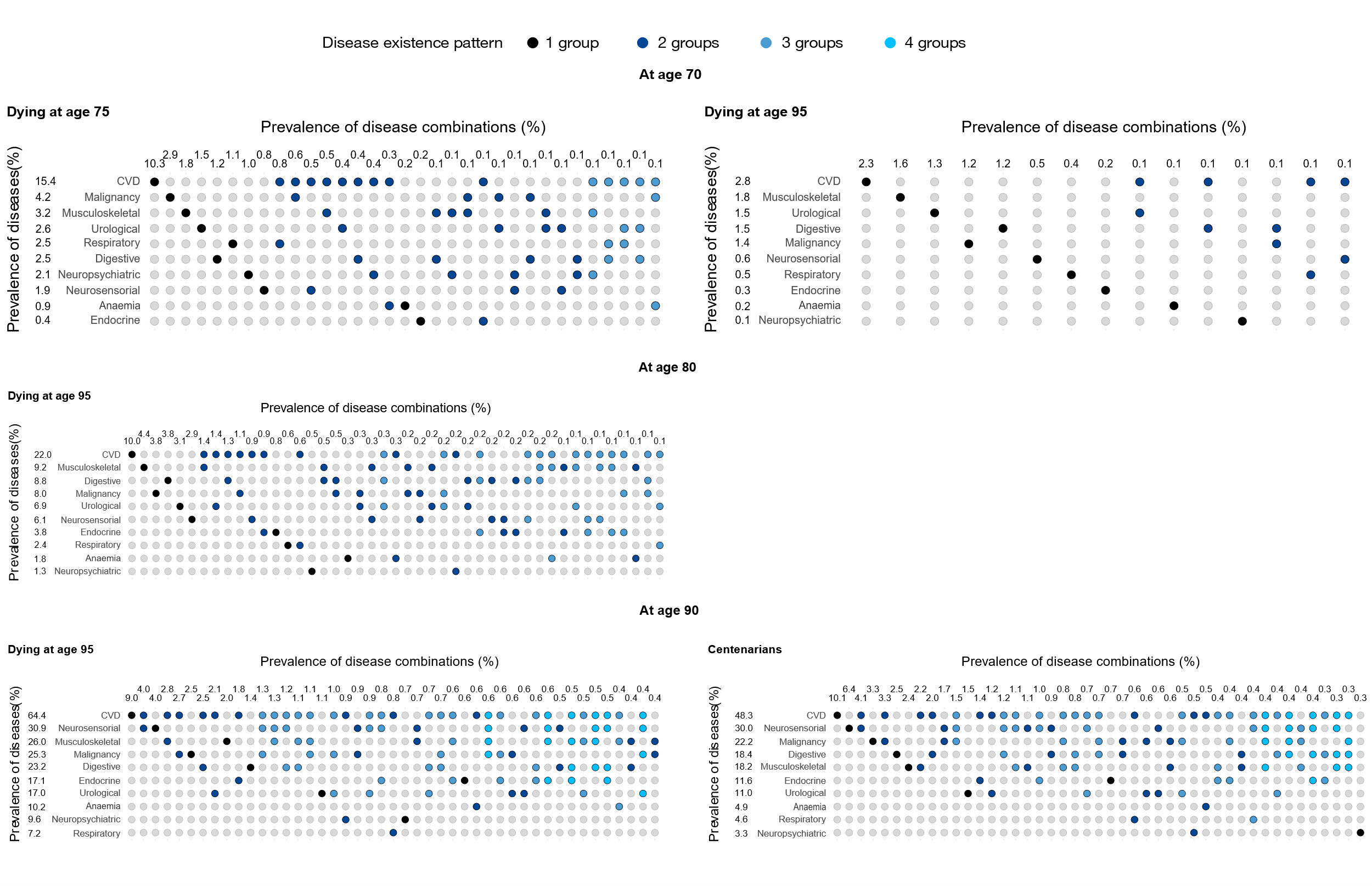


Figure S4 Prevalence of diseases (y-axis) and prevalence of disease combinations (x-axis) at ages 70, 80 and 90 for individuals with different life spans, birth cohorts 1920-1922, Sweden

Note: The x-axis represents the prevalence of the 45 most common disease existence patterns, ranked in descending order. Disease existence patterns are displayed using dots in different colours (single disease in black, 2 in dark blue, 3 in grey blue, 4 in sky blue). Only disease combination with a prevalence of 0.1% and higher are included in the figure.

Table S6 The share of having diseases confined in only one group at age 80

|  | **Centenarians (%)** | **Dying at age 85 (%)** | **Dying at age 95 (%)** | ***P (100,85)*** | ***P (100,95)*** |
| --- | --- | --- | --- | --- | --- |
| CVD | 55.5 | 34.6 | 45.5 | 2.20E-16 | 4.51E-05 |
| Musculoskeletal | 52.5 | 25.4 | 48.1 | 2.20E-16 | 0.231 |
| Digestive | 56.4 | 19.8 | 42.8 | 2.20E-16 | 0.0002 |
| Malignancy | 59.9 | 29.4 | 48.3 | 2.20E-16 | 0.002 |
| Neurosensorial | 50.4 | 23.9 | 47.1 | 4.15E-16 | 0.478 |
| Urological | 56.1 | 22.2 | 44.7 | 2.20E-16 | 0.013 |
| Endocrine | 33.0 | 8.3 | 21.9 | 4.23E-15 | 0.037 |
| Respiratory | 24.1 | 11.0 | 27.0 | 6.00E-03 | 0.809 |
| Anaemia | 25.0 | 9.3 | 18.9 | 0.001 | 0.494 |
| Neuropsychiatric | 44.4 | 19.2 | 37.8 | 0.003 | 0.700 |

Note: *P(age 100, 85)* represents comparison between centenarians and individuals dying at age 85. Similarly, *P (age 100, 95)* represents comparison between centenarians and individuals dying at age 95. *P*<0.05 was considered statistically significant.


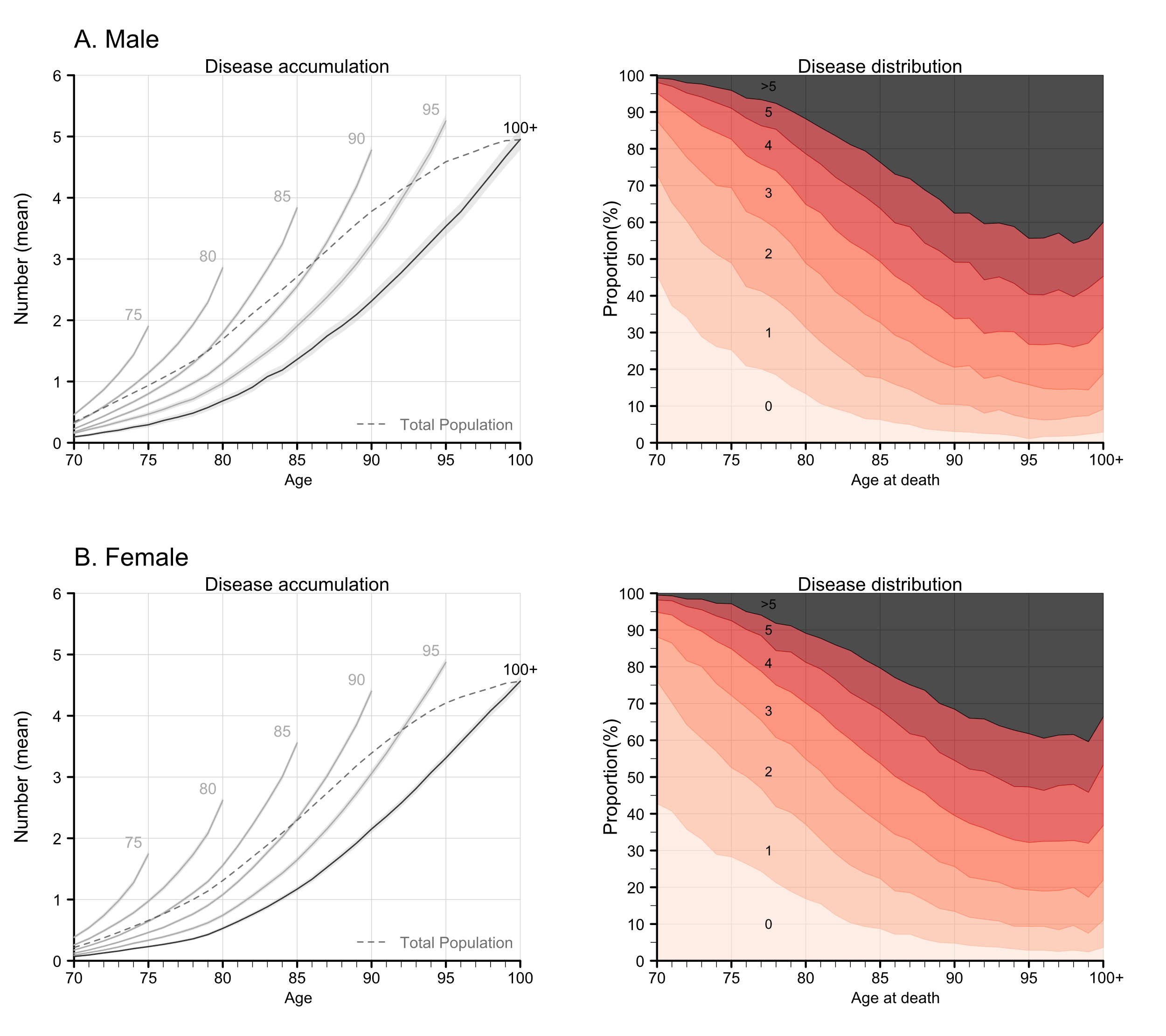


Figure S5 Disease accumulation from age 70 and proportion of individuals with 0 to >5 diseases by age at death for male (A) and female (B), birth cohorts 1920-1922, Sweden

Note: The numbers by each line in “Disease accumulation” represent age at death. The numbers in different coloured areas in “Disease distribution” represent numbers of diseases.


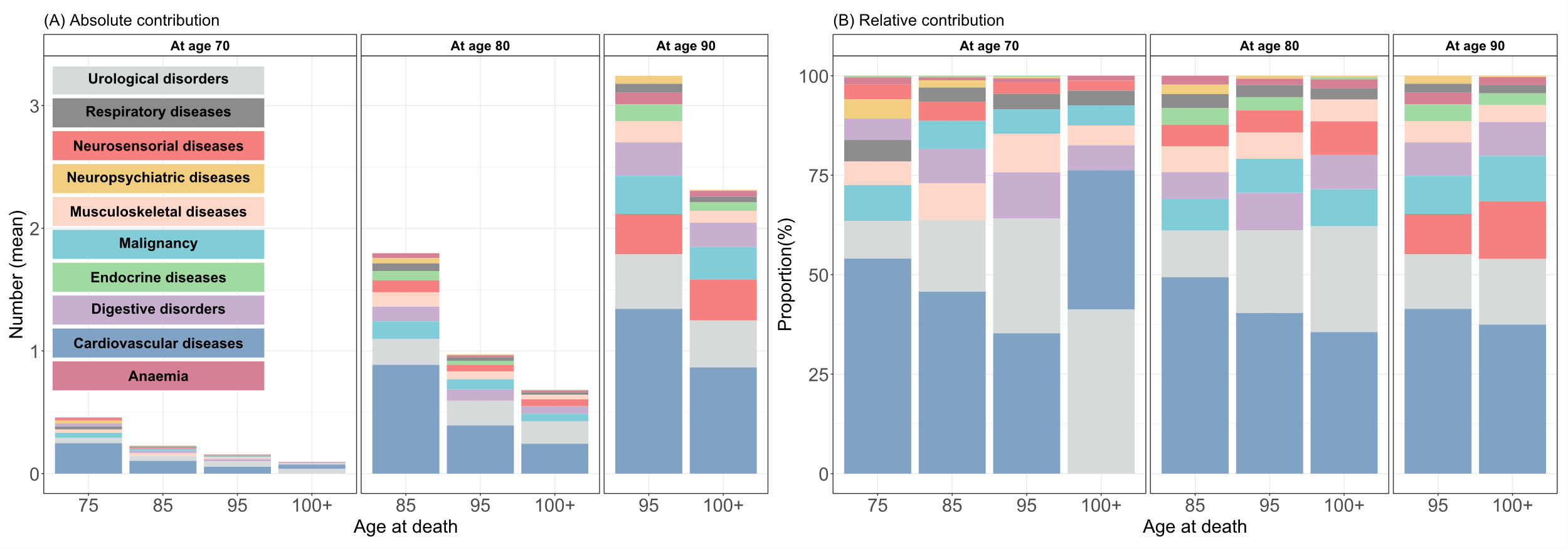


Figure S6 The absolute (A) and relative (B) contribution of different disease groups to the average number of diseases at ages 70, 80 and 90 for male with different lifespans (x-axis), birth cohorts 1920-1922, Sweden

Note: Diseases are presented in descending order, ranked from highest to lowest in terms of composition and proportion, from bottom to top.


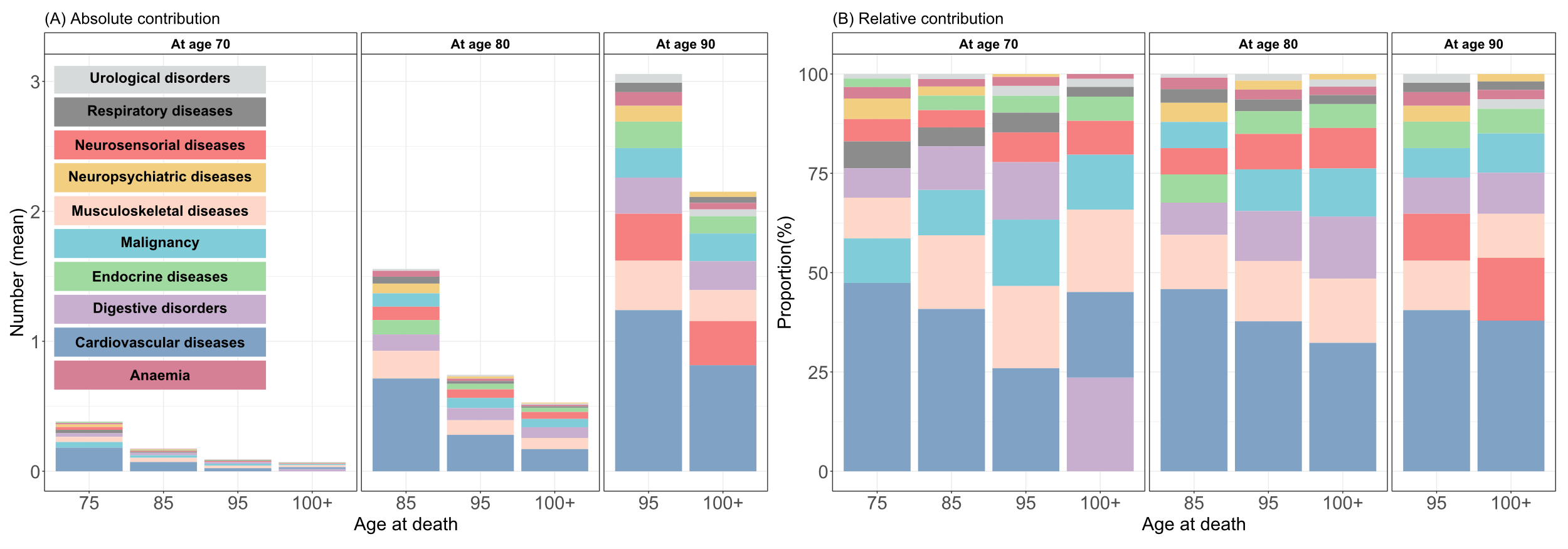


Figure S7 The absolute (A) and relative (B) contribution of different disease groups to the average number of diseases at ages 70, 80 and 90 for female with different lifespans (x-axis), birth cohorts 1920-1922, Sweden

Note: Diseases are presented in descending order, ranked from highest to lowest in terms of composition and proportion, from bottom to top.

Table S7 Changes in absolute contribution (mean number) of disease groups for male with different lifespans, birth cohorts 1920-1922, Sweden

|  | **Disease groups** | **Mean at age 70** | **Mean at age 80 (average change per age year from age 70 to 80)** | **Mean at age 90 (average change per age year from age 80 to 90)** |
| --- | --- | --- | --- | --- |
| Dying at age 75 | Endocrine | 0.002 | - | - |
|  | Anaemia | 0.008 | - | - |
|  | Neurosensorial | 0.017 | - | - |
|  | Neuropsychiatric | 0.022 | - | - |
|  | Digestive | 0.025 | - | - |
|  | Respiratory | 0.025 | - | - |
|  | Musculoskeletal | 0.027 | - | - |
|  | Malignancy | 0.041 | - | - |
|  | Urological | 0.043 | - | - |
|  | CVD | 0.248 | - | - |
|  | Total | 0.458 | - | - |
| Dying at age 85 | Endocrine | 0.001 | 0.075 (0.007) | - |
|  | Anaemia | 0.002 | 0.04 (0.004) | - |
|  | Neuropsychiatric | 0.004 | 0.043 (0.004) | - |
|  | Respiratory | 0.008 | 0.063 (0.006) | - |
|  | Neurosensorial | 0.011 | 0.097 (0.009) | - |
|  | Malignancy | 0.016 | 0.144 (0.013) | - |
|  | Digestive | 0.02 | 0.121 (0.010) | - |
|  | Musculoskeletal | 0.021 | 0.116 (0.010) | - |
|  | Urological | 0.041 | 0.21 (0.017) | - |
|  | CVD | 0.104 | 0.887 (0.078) | - |
|  | Total | 0.228 | 1.796 (0.157) | - |
| Dying at age 95 | Endocrine | 0.001 | 0.033 (0.003) | 0.137 (0.010) |
|  | Neuropsychiatric | 0.001 | 0.007 (0.001) | 0.065 (0.006) |
|  | Anaemia | 0.002 | 0.016 (0.001) | 0.095 (0.008) |
|  | Neurosensorial | 0.005 | 0.054 (0.005) | 0.328 (0.027) |
|  | Respiratory | 0.006 | 0.029 (0.002) | 0.073 (0.004) |
|  | Malignancy | 0.01 | 0.083 (0.007) | 0.311 (0.023) |
|  | Musculoskeletal | 0.015 | 0.064 (0.005) | 0.173 (0.011) |
|  | Digestive | 0.018 | 0.092 (0.007) | 0.273 (0.018) |
|  | Urological | 0.045 | 0.201 (0.016) | 0.445 (0.024) |
|  | CVD | 0.055 | 0.392 (0.034) | 1.343 (0.095) |
|  | Total | 0.158 | 0.971 (0.08) | 3.243 (0.227) |
| Centenarians | Endocrine | 0 | 0.004 (0.000) | 0.069 (0.007) |
|  | Neuropsychiatric | 0 | 0.002 (0.000) | 0.008 (0.001) |
|  | Anaemia | 0.001 | 0.016 (0.002) | 0.047 (0.003) |
|  | Neurosensorial | 0.002 | 0.058 (0.006) | 0.335 (0.028) |
|  | Respiratory | 0.004 | 0.019 (0.002) | 0.047 (0.003) |
|  | Malignancy | 0.005 | 0.064 (0.006) | 0.264 (0.020) |
|  | Musculoskeletal | 0.005 | 0.037 (0.003) | 0.098 (0.006) |
|  | Digestive | 0.006 | 0.059 (0.005) | 0.198 (0.014) |
|  | CVD | 0.034 | 0.243 (0.021) | 0.866 (0.062) |
|  | Urological | 0.04 | 0.182 (0.014) | 0.382 (0.020) |
|  | Total | 0.097 | 0.684 (0.059) | 2.314 (0.163) |

Note: Average change per age year between age 70 and 80 is calculated as: (mean number at age 80-mean number at age 70)/10.

Similarly, average change per age year between age 80 and 90 is calculated as: (mean number at age 90-mean number at age 80)/10.

Table S8 Changes in absolute contribution (mean number) of disease groups for female with different lifespans, birth cohorts 1920-1922, Sweden

|  | **Disease groups** | **Mean at age 70** | **Mean at age 80 (average change per age year from age 70 to 80)** | **Mean at age 90 (average change per age year from age 80 to 90)** | |
| --- | --- | --- | --- | --- | --- |
| Dying at age 75 | Urological | 0.004 | - | - |  |
|  | Endocrine | 0.008 | - | - |  |
|  | Anaemia | 0.011 | - | - |  |
|  | Neuropsychiatric | 0.02 | - | - |  |
|  | Neurosensorial | 0.022 | - | - |  |
|  | Respiratory | 0.026 | - | - |  |
|  | Digestive | 0.028 | - | - |  |
|  | Musculoskeletal | 0.039 | - | - |  |
|  | Malignancy | 0.043 | - | - |  |
|  | CVD | 0.182 | - | - |  |
|  | Total | 0.383 | - | - |  |
| Dying at age 85 | Urological | 0.002 | 0.015 (0.001) | - |  |
|  | Anaemia | 0.003 | 0.045 (0.004) | - |  |
|  | Neuropsychiatric | 0.004 | 0.075 (0.007) | - |  |
|  | Endocrine | 0.006 | 0.111 (0.011) | - |  |
|  | Neurosensorial | 0.008 | 0.103 (0.010) | - |  |
|  | Respiratory | 0.008 | 0.053 (0.005) | - |  |
|  | Digestive | 0.019 | 0.126 (0.011) | - |  |
|  | Malignancy | 0.02 | 0.103 (0.008) | - |  |
|  | Musculoskeletal | 0.032 | 0.212 (0.018) | - |  |
|  | CVD | 0.071 | 0.714 (0.064) | - |  |
|  | Total | 0.173 | 1.557 (0.138) | - |  |
| Dying at age 95 | Neuropsychiatric | 0.001 | 0.017 (0.002) | 0.122 (0.011) |  |
|  | Anaemia | 0.002 | 0.018 (0.002) | 0.105 (0.009) |  |
|  | Urological | 0.002 | 0.012 (0.001) | 0.068 (0.006) |  |
|  | Endocrine | 0.004 | 0.042 (0.004) | 0.203 (0.016) |  |
|  | Respiratory | 0.005 | 0.022 (0.002) | 0.072 (0.005) |  |
|  | Neurosensorial | 0.007 | 0.066 (0.006) | 0.362 (0.030) |  |
|  | Digestive | 0.013 | 0.093 (0.008) | 0.278 (0.019) |  |
|  | Malignancy | 0.015 | 0.078 (0.006) | 0.227 (0.015) |  |
|  | Musculoskeletal | 0.019 | 0.113 (0.009) | 0.38 (0.027) |  |
|  | CVD | 0.024 | 0.28 (0.026) | 1.24 (0.096) |  |
|  | Total | 0.092 | 0.741 (0.065) | 3.057 (0.232) |  |
| Centenarians | Neuropsychiatric | 0 | 0.007 (0.001) | 0.04 (0.003) |  |
|  | Anaemia | 0.001 | 0.011 (0.001) | 0.05 (0.004) |  |
|  | Urological | 0.001 | 0.009 (0.001) | 0.052 (0.004) |  |
|  | Respiratory | 0.002 | 0.012 (0.001) | 0.046 (0.003) |  |
|  | Endocrine | 0.004 | 0.032 (0.003) | 0.133 (0.010) |  |
|  | Neurosensorial | 0.006 | 0.054 (0.005) | 0.34 (0.029) |  |
|  | Malignancy | 0.01 | 0.064 (0.005) | 0.213 (0.015) |  |
|  | Musculoskeletal | 0.015 | 0.085 (0.007) | 0.238 (0.015) |  |
|  | CVD | 0.015 | 0.171 (0.016) | 0.816 (0.065) |  |
|  | Digestive | 0.017 | 0.083 (0.007) | 0.223 (0.014) |  |
|  | Total | 0.071 | 0.528 (0.046) | 2.151 (0.162) |  |

Note: Average change per age year between age 70 and 80 is calculated as: (mean number at age 80-mean number at age 70)/10.

Similarly, average change per age year between age 80 and 90 is calculated as: (mean number at age 90-mean number at age 80)/10.

Table S9 Changes in relative contribution (proportion) of disease groups for male with different lifespans, birth cohorts 1920-1922, Sweden

|  | **Disease groups** | **Proportion at age 70 (%)** | **Proportion at age 80 (%) (average change per age year from age 70 to 80)** | **Proportion at age 90 (%) (average change per age year from age 80 to 90)** |
| --- | --- | --- | --- | --- |
| Dying at age 75 | Endocrine | 0.4 | - | - |
|  | Anaemia | 1.7 | - | - |
|  | Neurosensorial | 3.8 | - | - |
|  | Neuropsychiatric | 4.8 | - | - |
|  | Digestive | 5.4 | - | - |
|  | Respiratory | 5.4 | - | - |
|  | Musculoskeletal | 5.9 | - | - |
|  | Malignancy | 9 | - | - |
|  | Urological | 9.5 | - | - |
|  | CVD | 54 | - | - |
| Dying at age 85 | Endocrine | 0.4 | 4.2 (0.38) | - |
|  | Anaemia | 0.8 | 2.2 (0.14) | - |
|  | Neuropsychiatric | 1.8 | 2.4 (0.06) | - |
|  | Respiratory | 3.6 | 3.5 (-0.01) | - |
|  | Neurosensorial | 4.8 | 5.4 (0.06) | - |
|  | Malignancy | 7 | 8 (0.1) | - |
|  | Digestive | 8.7 | 6.7 (-0.2) | - |
|  | Musculoskeletal | 9.3 | 6.5 (-0.28) | - |
|  | Urological | 18 | 11.7 (-0.63) | - |
|  | CVD | 45.7 | 49.4 (0.37) | - |
| Dying at age 95 | Endocrine | 0.3 | 3.3 (0.3) | 4.2 (0.09) |
|  | Neuropsychiatric | 0.3 | 0.7 (0.04) | 2 (0.13) |
|  | Anaemia | 1 | 1.6 (0.06) | 2.9 (0.13) |
|  | Neurosensorial | 2.9 | 5.5 (0.26) | 10.1 (0.46) |
|  | Respiratory | 3.9 | 3 (-0.09) | 2.3 (-0.07) |
|  | Malignancy | 6.1 | 8.6 (0.25) | 9.6 (0.1) |
|  | Musculoskeletal | 9.7 | 6.6 (-0.31) | 5.3 (-0.13) |
|  | Digestive | 11.7 | 9.5 (-0.22) | 8.4 (-0.11) |
|  | Urological | 28.8 | 20.7 (-0.81) | 13.7 (-0.7) |
|  | CVD | 35.3 | 40.4 (0.51) | 41.4 (0.1) |
| Centenarians | Endocrine | 0 | 0.5 (0.05) | 3 (0.25) |
|  | Neuropsychiatric | 0 | 0.4 (0.04) | 0.4 (0) |
|  | Anaemia | 1.2 | 2.3 (0.11) | 2 (-0.03) |
|  | Neurosensorial | 2.5 | 8.5 (0.6) | 14.5 (0.6) |
|  | Respiratory | 3.8 | 2.8 (-0.1) | 2 (-0.08) |
|  | Malignancy | 5 | 9.3 (0.43) | 11.4 (0.21) |
|  | Musculoskeletal | 5 | 5.5 (0.05) | 4.2 (-0.13) |
|  | Digestive | 6.2 | 8.6 (0.24) | 8.5 (-0.01) |
|  | CVD | 35 | 35.6 (0.06) | 37.4 (0.18) |
|  | Urological | 41.2 | 26.6 (-1.46) | 16.5 (-1.01) |

Note: Average change per age year between age 70 and 80 is calculated as: (proportion at age 80-proportion at age 70)/10.

Similarly, average change per age year between age 80 and 90 is calculated as: (proportion at age 90-proportion at age 80)/10.

Table S10 Changes in relative contribution (proportion) of disease groups for female with different lifespans, birth cohorts 1920-1922, Sweden

|  | **Disease groups** | **Proportion at age 70 (%)** | **Proportion at age 80 (%) (average change per age year from age 70 to 80)** | **Proportion at age 90 (%) (average change per age year from age 80 to 90)** |
| --- | --- | --- | --- | --- |
| Dying at age 75 | Urological | 1.1 | - | - |
|  | Endocrine | 2.1 | - | - |
|  | Anaemia | 2.9 | - | - |
|  | Neuropsychiatric | 5.2 | - | - |
|  | Neurosensorial | 5.6 | - | - |
|  | Respiratory | 6.8 | - | - |
|  | Digestive | 7.4 | - | - |
|  | Musculoskeletal | 10.3 | - | - |
|  | Malignancy | 11.2 | - | - |
|  | CVD | 47.4 | - | - |
|  | Urological | 1.3 | 0.9 (-0.04) | - |
| Dying at age 85 | Anaemia | 1.9 | 2.9(0.1) | - |
|  | Neuropsychiatric | 2.3 | 4.8 (0.25) | - |
|  | Endocrine | 3.7 | 7.1 (0.34) | - |
|  | Neurosensorial | 4.3 | 6.6 (0.23) | - |
|  | Respiratory | 4.8 | 3.4 (-0.14) | - |
|  | Digestive | 11 | 8.1 (-0.29) | - |
|  | Malignancy | 11.4 | 6.6 (-0.48) | - |
|  | Musculoskeletal | 18.6 | 13.6 (-0.5) | - |
|  | CVD | 40.9 | 45.9 (0.5) | - |
| Dying at age 95 | Neuropsychiatric | 0.7 | 2.3 (0.16) | 4 (0.17) |
|  | Anaemia | 2.2 | 2.5 (0.03) | 3.4 (0.09) |
|  | Urological | 2.5 | 1.7 (-0.08) | 2.2 (0.05) |
|  | Endocrine | 4.2 | 5.7 (0.15) | 6.6 (0.09) |
|  | Respiratory | 5 | 2.9 (-0.21) | 2.3 (-0.06) |
|  | Neurosensorial | 7.5 | 8.9 (0.14) | 11.8 (0.29) |
|  | Digestive | 14.5 | 12.6 (-0.19) | 9.1 (-0.35) |
|  | Malignancy | 16.7 | 10.5 (-0.62) | 7.4 (-0.31) |
|  | Musculoskeletal | 20.7 | 15.2 (-0.55) | 12.4 (-0.28) |
|  | CVD | 25.9 | 37.7 (1.18) | 40.6 (0.29) |
| Centenarians | Neuropsychiatric | 0 | 1.4 (0.14) | 1.9 (0.05) |
|  | Anaemia | 1.2 | 2.1 (0.09) | 2.3 (0.02) |
|  | Urological | 2 | 1.8 (-0.02) | 2.4 (0.06) |
|  | Respiratory | 2.4 | 2.3 (-0.01) | 2.1 (-0.02) |
|  | Endocrine | 6.1 | 6 (-0.01) | 6.2 (0.02) |
|  | Neurosensorial | 8.5 | 10.2 (0.17) | 15.8 (0.56) |
|  | Malignancy | 13.8 | 12.1 (-0.17) | 9.9 (-0.22) |
|  | Musculoskeletal | 20.7 | 16.2 (-0.45) | 11.1 (-0.51) |
|  | CVD | 21.5 | 32.3 (1.08) | 37.9 (0.56) |
|  | Digestive | 23.6 | 15.6 (-0.8) | 10.4 (-0.52) |

Note: Average change per age year between age 70 and 80 is calculated as: (proportion at age 80-proportion at age 70)/10.

Similarly, average change per age year between age 80 and 90 is calculated as: (proportion at age 90-proportion at age 80)/10.
